# 'Unite and conquer': enhanced prediction of protein subcellular localization by integrating multiple specialized tools

**DOI:** 10.1186/1471-2105-8-420

**Published:** 2007-10-29

**Authors:** Yao Qing Shen, Gertraud Burger

**Affiliations:** 1 Robert Cedergren Center for Bioinformatics and Genomics, Biochemistry Department, Université de Montréal, 2900 Edouard-Montpetit, Montreal, QC, H3T 1J4, Canada

## Abstract

**Background:**

Knowing the subcellular location of proteins provides clues to their function as well as the interconnectivity of biological processes. Dozens of tools are available for predicting protein location in the eukaryotic cell. Each tool performs well on certain data sets, but their predictions often disagree for a given protein. Since the individual tools each have particular strengths, we set out to integrate them in a way that optimally exploits their potential. The method we present here is applicable to various subcellular locations, but tailored for predicting whether or not a protein is localized in mitochondria. Knowledge of the mitochondrial proteome is relevant to understanding the role of this organelle in global cellular processes.

**Results:**

In order to develop a method for enhanced prediction of subcellular localization, we integrated the outputs of available localization prediction tools by several strategies, and tested the performance of each strategy with known mitochondrial proteins. The accuracy obtained (up to 92%) surpasses by far the individual tools. The method of integration proved crucial to the performance. For the prediction of mitochondrion-located proteins, integration via a two-layer decision tree clearly outperforms simpler methods, as it allows emphasis of biologically relevant features such as the mitochondrial targeting peptide and transmembrane domains.

**Conclusion:**

We developed an approach that enhances the prediction accuracy of mitochondrial proteins by uniting the strength of specialized tools. The combination of machine-learning based integration with biological expert knowledge leads to improved performance. This approach also alleviates the conundrum of how to choose between conflicting predictions. Our approach is easy to implement, and applicable to predicting subcellular locations other than mitochondria, as well as other biological features. For a trial of our approach, we provide a webservice for mitochondrial protein prediction (named YimLOC), which can be accessed through the AnaBench suite at http://anabench.bcm.umontreal.ca/anabench/. The source code is provided in the Additional File 2.

## Background

The eukaryotic cell is highly organized: various biological processes are associated with specialized subcellular structures (such as protein export across the cell membrane), or confined to particular compartments (e.g., respiration in mitochondria). Subcellular location provides important clues about a protein's function and this knowledge is therefore used to assist in the annotation of newly discovered or sequence-inferred proteins. On the other hand, the location of proteins with known function unravels where the corresponding biological processes take place and how they are connected amongst each other. Proteomics and microscopic detection of tagged or labelled proteins are powerful experimental approaches for determining protein localization. However, for most species, these approaches are costly in time and expense, and so there is a need for *in silico *prediction. A plethora of bioinformatic prediction methods have been developed in the past [1-21], and a dozen or so computational tools are publicly available (for a review see [22]). Most of these tools employ machine learning methods, i.e., they learn location-specific sequence features from known examples, and then extrapolate the learned rules to make predictions for proteins of unknown locations.

The targeting peptide, a conserved sequence motif usually located at the N-terminus of proteins, is a widely used sequence feature to identify a protein's location within the cell. This signal interacts with the import machineries of organelles such as mitochondria, chloroplasts and the endoplasmic reticulum. A number of tools use this signal for identifying proteins imported into organelles, notably **MitoProt **[23], **TargetP **[24], **iPSORT **[25], **Protein Prowler **[26], **Signal-CF **[27], and **Predotar **[28]. However, some organelle-imported proteins lack a N-terminal targeting peptide (e.g., the ADP/ATP carrier that is embedded in the inner mitochondrial membrane [29]) and therefore remain undetected by the tools above. In addition, application of these tools for genome-sequence-inferred proteins is limited, because the N-terminus of hypothetical proteins is often uncertain.

Another approach to identifying protein localization is based on sequence similarity with proteins of known location. For instance, a protein which shares a high similarity with a mitochondrial NADH:ubiquinone oxidoreductase subunit is very likely located in mitochondria. Sequence similarity combined with text annotation is used, for example, by the web-server 'Proteome Analyst Specialized Subcellular Localization Server' (**PASUB**) [30]. **PSLT **[31] predicts protein localization by searching for particular protein motifs and membrane domains. The underlying assumption is that proteins belonging to the same compartment share common domains. Both sequence-similarity-based and domain-based predictions have the limitation of depending on the existence of known homologs or known domains.

Several prediction tools do not rely on sequence similarity to known proteins or domains, but instead exploit a protein's amino acid composition and biochemical properties. **Subloc **[32], for instance, classifies proteins according to amino acid frequency, while **CELLO **[33] uses ungapped and gapped amino acid pair composition.

Certain tools combine several inherent sequence features and some also include textual information. For example, **ESLpred **[34] uses n-peptide composition and physicochemical properties, together with PSI-BLAST results. **pTARGET **[35] calculates scores based on the occurrence pattern of Pfam domains [36] and amino acid composition. **SherLoc **[37] exploits amino acid composition, targeting peptides, and motifs, as well as annotation and text description drawn from the literature or SwissProt entries.

It has been shown before that combining various prediction methods often yields better accuracy than the individual methods [38]. In fact, several of the above mentioned tools integrate different classifiers. **CELLO **[33], for instance, employs a two-level support vector machine (SVM) classification system. The first level builds individual SVM classifiers, one each for n-peptide composition, gapped-dipeptide composition, and so on. Each of these classifiers generates a probability distribution, which is then processed by a second-level SVM to calculate the final probability for a protein to belong in a certain subcellular location. The second-level SVM achieves a notably higher accuracy than the individual first-level classifiers. Similarly, **SherLoc **[37] uses the output vectors of different sequence-based classifiers and a text-based classifier as input for the final SVM classifier. An alternative approach builds Bayesian classifiers based on Markov chains, and constructs a hierarchical ensemble of these classifiers [39].

Each of the available localization prediction tools (subsequently referred to as LOC-tools) has different strength, and no tool is clearly and globally optimal. Any given LOC-tool performs well on certain data but poorly on others, and often predictions by different tools disagree (see examples in Table 1). This is not surprising, because LOC-tools employ different machine learning algorithms, sequence features, and training data.

**Table 1:** Examples of conflicting results from individual prediction tools

**Sequence ID**^1^	**Experimentally verified location**	**Predictions of mitochondrial location by individual LOC-tools**^2,3^								
		
		TargetP	Subloc	pTARGET	SherLoc	Predotar	MitoProt	CELLO	PProwler	PASUB
YOR297C	Mitochondria	mit	mit	mit	non	non	mit	non	non	mit
YDR378C	Nucleus	mit	mit	non	non	non	mit	mit	non	non

^1 ^The example sequences are retrieved from the yeast genome database [52]

^2 ^For references see text

^3 ^"mit", predicted as mitochondrial protein; "non", predicted as non-mitochondrial protein

This report introduces a comprehensive and simple system for protein location prediction. Following the maxim '*unite *and conquer', our approach combines the complementary strengths of existing prediction methods. Using the example of mitochondrial location, we integrated heterogeneous localization predictors by different strategies, tested performance with known data and selected the most efficient way of integration. The presented methodology is readily applicable to proteins from subcellular locations other than mitochondria, and even to the prediction of other biological features for which multiple, heterogeneous tools exist.

## Results

**Figure 1:**
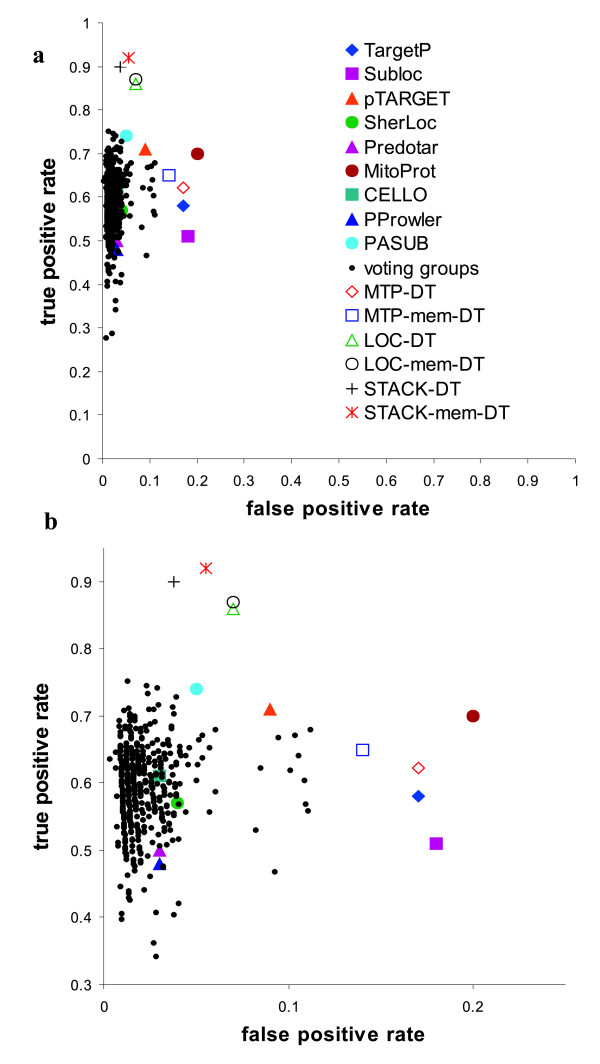
**Prediction performance of individual and integrated tools on yeast mitochondrial proteins**. **Filled symbols**: individual LOC-tools; **Dots**: voting groups (tools integrated by majority-win voting); **Open symbols**: decision trees. The desired results are located in the top left of the plot area, representing high true positive rate and low false positive rate. **a**, the result shown at full scale. **b**, the zoom-in of the region with false positive rate 0~0.25, and true positive rate 0.3~0.95.

As described in the Method section, we collected ~1,000 yeast proteins, ~1,000 *Arabidopsis *proteins, and ~3,000 human proteins of known subcellular location. Figure 1 shows the performance of nine individual LOC-tools on these data sets: TargetP, Subloc, SherLoc, pTARGET, Predotar, PProwler, PASUB, MitoProt, and CELLO. In the subsequent step, the predictions of these heterogeneous tools were integrated by different strategies. We employed the same procedure for all three datasets. Here, we show the results for yeast; those for *Arabidopsis *and human are given in Additional File 1.

### Integration of LOC-tool predictions by grouping and majority-win voting

We formed 502 different groups ("voting groups") from nine individual LOC-tools. The predictions of the tools within each group are integrated by majority-win voting (see Methods section). Figure 1 (dots) shows that the performance on mitochondrial proteins varies greatly among the groups (see also Additional File 1: Figures S1 – S2). While the False Positive Rate (FPR) is generally low (< 0.05), the True Positive Rate (TPR) varies from 0.26 to 0.75. The best result is produced by the voting group pTARGET+PASUB+CELLO (TPR: 0.75, FPR: 0.02), but PASUB alone performs nearly as well (TPR: 0.74, FPR: 0.05). Thus, the gain of integration by majority-win voting is only moderate.

### Integration of LOC-tool predictions by decision tree

**Figure 2:**
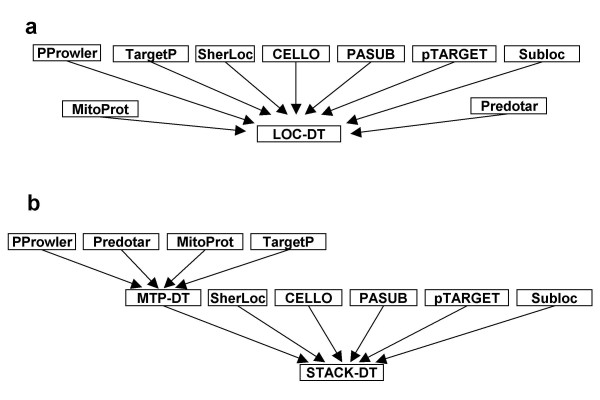
**Integration of heterogeneous prediction tools by decision trees**. **a**, The LOC-DT was built with outputs from nine LOC-tools. **b**, The MTP-DT was built with outputs from four tools whose prediction is based on the mitochondrial targeting peptide. The output of MTP-DT, together with the outputs of five other LOC-tools, was used to construct the STACK-DT.

For integration by decision trees, we took the predictions of the LOC-tools as input to construct classifiers by the C4.5 algorithm [40]. A total of six different decision trees were built as summarized in Table 2. First, outputs of all LOC-tools were employed as equivalent attributes. The resulting decision tree (referred to as LOC-DT, Figure 2a) recognizes mitochondrial proteins with an average TPR of 0.86 and FPR of 0.07, as evaluated by the ten-fold cross validation test (Figure 1, open symbols; Additional File 1: Figures S1 – S2). Note that the decision tree classifiers did not retain all the LOC-tools provided in the training process. The elimination of a given tool is due either to redundancy or to low accuracy such that its inclusion would cause performance to deteriorate.

**Table 2:** Decision trees built in this study and the individual tools employed to construct each tree^a^

Decision trees	LOC-tools									MEM-tools			
	
	TargetP	Predotar	MitoProt	PProwler	CELLO	Subloc	pTARGET	SherLoc	PASUB	Phobius	TMHMM	HMMTOP	SOSUI
LOC-DT	X	X	X	X	X	X	X	X	X				
MTP-DT	X	X	X	X									
STACK-DT	MTP-DT				X	X	X	X	X				
LOC-mem-DT	X	X	X	X	X	X	X	X	X	X	X	X	X
MTP-mem-DT	X	X	X	X						X	X	X	X
STACK-mem-DT	MTP-DT				X	X	X	X	X	X	X	X	X

^a ^"X", if the tool is included in the decision tree listed in the leftmost column (for the references see text)

Second, we introduced biological expert knowledge into the construction of decision trees. The mitochondrial targeting peptide (MTP) is a feature exclusive to mitochondrial proteins, and four LOC-tools rely on it to make predictions. In order to better exploit this feature, we implemented a decision tree integrating four MTP-based tools used in this study, notably TargetP, MitoProt, Predotar and PProwler. The output of this decision tree (referred to as MTP-DT) was then combined with the other five tools by constructing a stacked decision tree (STACK-DT; Figure 2b). As expected, stacking results in a major performance increase with a TPR of 0.9 and FPR of 0.04.

### Effect of including transmembrane domain prediction tools

**Figure 3:**
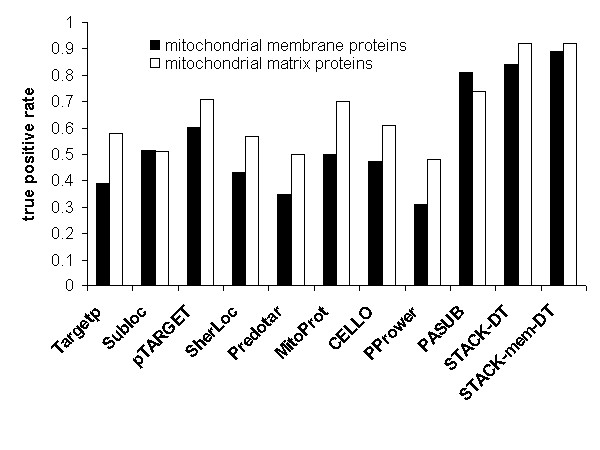
**Prediction performance of individual and integrated tools on yeast mitochondrial membrane and matrix proteins**. Loc-tools recognize mitochondrial membrane proteins less efficiently than matrix proteins. The effectiveness of PASUB is due to the fact that it exploits annotations and that the portion of *annotated *mitochondrial membrane proteins is higher compared to matrix proteins.

We realized that LOC-tools recognize membrane proteins less efficiently than matrix proteins (Figure 3). To alleviate this shortcoming, we integrated the LOC-tools with four additional tools that predict transmembrane domains (MEM-tools), i.e., **Phobius **[41], **TMHMM **[42], **HMMTOP **[43], and **SOSUI **[44]. The decision trees incorporating MEM-tools and LOC-tools are termed LOC-mem-DT, MTP-mem-DT and STACK-mem-DT (see Table 2).

Figure 3 shows that the integration of MEM-tools with LOC-tools clearly improves the recognition of mitochondrial membrane proteins. It should be noted that such improvement is not directly reflected in the overall performance, because mitochondrial membrane proteins account for only ~10% of our dataset.

Out of the six decision trees described above, STACK-mem-DT displays by far the best performance. Compared with the best individual LOC-tool and the best voting group (see above), STACK-mem-DT excels particularly in its high TPR (Table 3). This result was obtained from a dataset clustered at a cutoff of 80% sequence identity (data_C80). We repeated these experiments with datasets clustered more stringently at a 25% sequence identity cutoff (data_C25, Additional File 1: Table S2). The outcome was essentically the same as with data_C80 (Additional File 1: Table S3), which means that the good performance of STACK-mem-DT is not a result of data redundancy.

**Table 3:** Performance^1 ^of the best predictors for the three different prediction schemes

**Classes**^2^		**Individual tool (PASUB)**			**Combination of tools by voting**^3^			**Decision tree classifier (STACK-mem-DT)**		
		
		TPR	FPR	ACC	TPR	FPR	ACC	TPR	FPR	ACC
Yeast	Mit	0.74	0.05	0.69	0.75	0.02	0.84	0.92	0.05	0.95
	Non	0.65	0.06		0.99	0.20		0.97	0.05	
*Arabidopsis*	Mit	0.75	0.09	0.81	0.67	0.07	0.88	0.87	0.12	0.94
	Non	0.83	0.05		0.95	0.09		0.96	0.04	
Human	Mit	0.87	0.09	0.68	0.88	0.01	0.97	0.90	0.02	0.99
	Non	0.65	0.02		0.98	0.02		0.99	0.01	

^1 ^TPR: true positive rate; FPR: false positive rate; ACC: accuracy (all correctly predicted instances/all instances)

^2 ^Mit: mitochondrial proteins; Non: proteins of other subcellular locations

^3 ^The best combination of tools is pTARGET+PASUB+CELLO for yeast data, PASUB+MitoPort+CELLO for *Arabidopsis *data, and pTARGET +SherLoc+ PASUB for human data

We were concerned that this superior performance was caused by a 20~50% overlap of our yeast data and the training data of individual LOC-tools. Therefore, we constructed a data subset, excluding proteins present in, or similar to, the training data of any LOC-tool, to build new decision trees. The result shows that the superior performance of STACK-mem-DT over both individual LOC-tools and majority-win voting is retained with this subset (Additional File 1: Figure S3).

**Figure 4:**
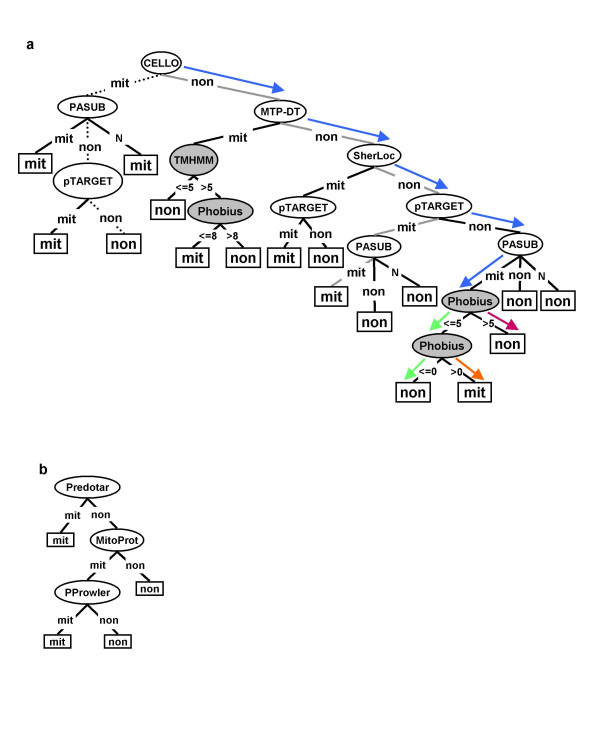
**Decision tree topology for the prediction of mitochondrial proteins**. **a**, STACK-mem-DT; **b**, MTP-DT. The trees were built by C4.5 (see Methods). Each oval represents a prediction tool. **Filled ovals **represent transmembrane domain predictors. **Rectangle **represents a decision: "mit" for mitochondrial proteins and "non" for proteins of other subcellular locations. If a tool predicts the query protein as a mitochondrial protein, the branch (edge) is labeled "mit"; otherwise "non". If PASUB makes no prediction, the branch is labeled "N". Several decision-making paths are highlighted, as follows: **Dotted line**: for non-mitochondrial protein YDR378C. **Grey line**: for mitochondrial protein YOR297C. **Blue arrow**: the common path for three differently localized proteins: mitochondrial (YIL065C), plasma membrane (YBR069C) and nuclear (YLL022C). **Orange arrow**: for mitochondrial protein YIL065C. **Red arrow**: for non-mitochondrial protein YBR069C. **Green arrow**: for non-mitochondrial protein YLL022C.

To dissect how STACK-mem-DT makes its predictions, we followed the specific decision paths of the mitochondrial and nuclear proteins listed in Table 1, proteins that individual tools predict conflictingly. The mitochondrial protein follows a path down to SherLoc with all three predictions being wrong (Figure 4a). But in the end, the decision tree recognizes the mitochondrial location due to the two correct predictions made by pTARGET and PASUB. Similarly, the nuclear protein is first wrongly classified by CELLO, but the subsequent steps of the path identify its true location.

Finally, we inspected the paths of three other proteins, constituents of the mitochondrial outer membrane, the plasma membrane and the nucleus, respectively. All of these proteins cannot be distinguished by the individual LOC-tools (Table 4), nor by trees without MEM-tools. STACK-mem-DT correctly classifies all three proteins due to the final two steps in the tree that employ MEM-tools (Figure 4a, coloured line).

**Table 4:** Example proteins used for decision tracing

**Sequence ID**^1^	**Experimentally verified location**	**Predictions of mitochondrial location by individual LOC-tools**^2,3^									**Predicted number of transmembrane domains**^2,3^			
		
		TargetP	Subloc	pTARGET	SherLoc	Predotar	MitoProt	CELLO	PProwler	PASUB	Phobius	TMHMM	HMMTOP	SOSUI
YIL065C	Mitochondrial outer membrane	non	non	non	non	non	non	non	non	mit	1	1	1	1
YBR069C	Plasma membrane	non	non	non	non	non	non	non	non	mit	12	12	12	12
YLL022C	Nucleus	non	non	non	non	non	non	non	non	mit	0	0	0	0

^1 ^The sequences are retrieved from the yeast genome database [52]

^2 ^For references see text

^3 ^"mit", predicted as mitochondrial protein; "non", predicted as non-mitochondrial protein

### Implementation

STACK-mem-DT was implemented as a webservice, YimLOC, accessible via the public bioinformatics workbench AnaBench [45]. The current version takes the prediction results from individual tools as input, and outputs the prediction for a protein to be mitochondrion-localized or not. For thorough analyses, we recommend that users build the decision tree on their local computer, with their own training data and choice of individual LOC-tools. The source code is available under the GNU licence.

## Discussion

The purpose of this study was to enhance prediction accuracy by integrating the available subcellular localization prediction tools. Successful integration of specialized tools takes advantage of their complementary strengths, which are drawn from three sources: the different sequence features the tools exploit, the different computational algorithms they employ, and the different training sets they are built from.

### Integration by decision tree outperforms group voting

The best performance obtained from majority-win voting of LOC-tool groups shows almost the same TPR as the best individual LOC-tool (PASUB in this case), with a slightly lower FPR. Some of the voting groups yield even lower TPRs than individual LOC-tools. In contrast, decision tree classifiers built from the ensemble of LOC-tools all outperform the individual tools as well as any of the majority-win voting combinations (see Figure 1. Note that MTP-DT and MTP-mem-DT are special cases as they were given only a subset of LOC-tools for training.). The most effective of the presented integrative predictors is STACK-mem-DT, which exceeds by far the performance of the best LOC-tool (TPR of 0.92 compared to 0.75, with the same FPR of 0.05; Table 3). Yet, for fairness, it should be stressed that many of the tools have been developed with the aim of predicting multiple locations, while we optimize here mitochondrial location.

A fair and rigorous comparison of YimLOC with all other prediction methods should use the same test data, as we did for the comparison of YimLOC with nine LOC-tools shown in Figure 1, and in Additional File 1: Figures S1 – S2. Unfortunately, this is not feasible for some prediction methods because of several reasons: the training data are not provided; there are no webservices or software distributions available; the webservices are available but not tuned for large-scale predictions.

Among the various machine leaning methods, we chose here decision trees for integration because they have the advantage that they allow tracing back how the predictions are made, and thus may provide a biological meaningful interpretation of the predictions. Note that for the more complex problem of predicting proteins targeted to multiple subcellular locations [4-6], neural network or Naïve Bayes would be more appropriate than decision trees, because they allow handling of prediction probabilities in a flexible manner.

### Trade-off between sensitivity and specificity

For any given prediction method, an increase of the TPR is usually accompanied by an increase of the FPR. How to balance the two rates depends on the purpose of the prediction. If biologists wish to identify *all *mitochondrial proteins from a whole genome sequence, they should choose a prediction method with highest TPR (in this study the STACK-mem-DT). On the other hand, if the purpose is to determine the subcellular localization of a few candidate proteins of interest, a prediction method with lowest FPR should be favoured (in this study the combination of pTARGET+PASUB+CELLO).

### Making use of prior biological knowledge

During decision tree construction, LOC-tools are retained if they have a good overall performance on the training data. In this process, all tools (and therefore the sequence features exploited) are considered of equal importance. To further enhance performance, we put more emphasis on certain tools based on domain-specific knowledge. In particular, the mitochondrial targeting peptide (MTP) is specific to proteins imported into mitochondria, but not all mitochondrial proteins possess one. Therefore, a tool that recognizes mitochondrial proteins based on the presence of MTP has high specificity (a protein with MTP is reliably targeted to mitochondria), but low sensitivity (mitochondrial proteins without MTP cannot be recognized). We employed four MTP-based tools in this study. Yet, LOC-DT retained only one of them, although the other three tools may be complementary in recognizing the various instances.

Since the targeting peptide is known to be an important determinant of protein localization, but not necessarily rewarded by decision trees, we modified the training process to make use of this external knowledge. This was achieved by a two-layer decision tree (STACK-DT, see Figure 2b). Indeed, STACK-DT performes significantly better than LOC-DT (see Figure 1, "+"), testifying to the value of incorporating expert knowledge in decision tree construction.

### Inclusion of transmembrane domain prediction

We observed that LOC-tools often misclassified mitochondrial membrane proteins (Figure 3). This may be due to several reasons: (i) the training sets of some tools do not include mitochondrial membrane proteins (e.g., Subloc); (ii) mitochondrial membrane proteins typically lack a targeting peptide, while MTP-based tools rely on the presence of this signal [46]; and (iii) tools based on amino acid composition and physicochemical properties may confuse mitochondrial membrane proteins with membrane proteins from other subcellular compartments. We have addressed these limitations by building decision tree classifiers that integrate predictions of both subcellular localization and transmembrane domains. In fact, information on the number of such domains boosts recognition of mitochondrial membrane proteins from 81% to 89% (Figure 3).

## Conclusion

This study devises a simple, practical and highly effective approach to exploiting complementary bioinformatics tools by integration through machine learning. Using mitochondrial location as a test case, we observe that tool integration with decision trees significantly improves prediction accuracy compared to individual tools or their simple combination. Inclusion of biological expert knowledge in machine learning further enhances the performance. Particularly improved is prediction of membrane proteins, which is notoriously difficult. Further, our approach alleviates the conundrum of how to choose between conflicting predictions from different LOC-tools. The methodology is easy to implement and applicable to the prediction of other biological feature for which multiple, heterogeneous tools exist.

## Methods

### Data set

Protein sequences from yeast in Swiss-Prot release 50.3 were selected by the following criteria: 1) they are encoded in the nucleus; 2) their subcellular location is experimentally verified; and 3) the localization annotation is not ambiguous (i.e., terms like "probable" or "possible" are absent from their annotation of subcellular localization). In addition, we retrieved 522 yeast mitochondrial protein sequences from MITOP2 [47], a manually curated database of nucleus-encoded mitochondrial proteins with experimental evidence. Sequences having identities over 80% were clustered by Cd-hit [48] to reduce data redundancy. The final yeast dataset contains 503 mitochondrial and 872 non-mitochondrial proteins.

In a similar way, *Arabidopsis *and human protein sequences from Swiss-Prot were collected. The *Arabidopsis *dataset was enriched by sequences from AMPDB [49], a database for *Arabidopsis *mitochondrial proteins. After being clustered with 80% sequence identity, 193 mitochondrial and 608 non-mitochondrial proteins constitute the *Arabidopsis *dataset. The human dataset contains 353 mitochondrial and 2,679 non-mitochondrial proteins.

In addition, we further clustered the three datasets (yeast, *Arabidopsis*, and human) with the threshold of 25% sequence identity to build more stringent datasets (Additional File 1: Table S2).

To compile a dataset which does not overlap with the training data of the LOC-tools employed (see Table 2), we searched our yeast dataset against the training data of the nine LOC-tools with BLAST. A protein was removed from the yeast data if it had >80% identity to a protein in the training set of any LOC-tool. The remaining proteins constitute a non-overlapping subset of yeast data, which contains 190 mitochondrial and 344 non-mitochondrial proteins.

### Integration of heterogeneous tools

#### a Prediction by individual tools

We selected nine prediction tools for subcellular localization: TargetP [24], Subloc [32], SherLoc [37], pTARGET [35], Predotar [28], Protein Prowler (PProwler) [26], PASUB [30], MitoProt [23], and CELLO [33]. The selection was based on the diversity of the algorithms and the sequence features they employ. These tools were used as base-level classifiers, whose prediction results were combined to build new classifiers. Prediction results from most tools were obtained via web services. The only exception is MitoProt, which has been installed and run locally.

#### b Consistent representation of the output from heterogeneous LOC-tools

LOC-tools output a categorical prediction (mitochondria, cytoplasm, nucleus, etc.) for each query sequence. Predictions were converted to "mit" for mitochondrial location and "non" otherwise. A special case is PASUB, which makes no predictions for proteins that lack significant similarity to known sequences. In these cases, we issued "N".

Together with the categorical prediction, LOC-tools also output a positive numerical value indicating the confidence of prediction. The range of numerical values differs among LOC-tools. Intuitively, numerical encoding seems advantageous, since it reflects the confidence that LOC-tools have in their predictions. However, it also may introduce a hidden bias in the integration, because the various tools evaluate and measure confidence differently (Additional File 1: Table S1). For example, CELLO outputs a score (for example 2.064) to show the reliability that a protein is affiliated with each of 12 subcellular locations. In contrast, pTARGET distinguishes nine locations, and outputs the confidence value in the form of percentage (for example 98%). Since it is not straightforward to consolidate the particular confidence factors of the various LOC-tools, we decided to use categorical encoding.

#### c Integration of LOC-tools by grouping and voting

For nine LOC-tools, with group size from two to nine, there were a total of 502 different groups. Within each group, predictions of individual LOC-tools were combined with a majority-win voting scheme. A given sequence was regarded as a mitochondrial protein, if more than half of the combined tools assigned it to mitochondria. No prediction was made if there was a tie.

#### d Integration of LOC-tools by decision tree

For building decision trees, we used J4.8, a program based on the C4.5 algorithm [40], available in the Weka package [50]. Default parameters were employed. The individual LOC-tools and MEM-tools were used as attributes of input data, and the prediction results of each tool as attribute values.

The decision trees were evaluated by a ten-fold cross validation test, where the data set was equally divided into ten parts. Nine parts were combined to form the training set for building the decision tree, which was then evaluated by the remaining part. The process was repeated ten times. Alternatively, jackknife test can be employed for examining the power of a prediction method [1-3]. Although jackknife test is deemed the most rigorous and objective [51], it is time consuming, particularly for large datasets. Therefore, 10-fold cross validation is a good and wildly adopted alternative.

The performance of each prediction method was measured as true positive rate and false positive rate, where

true positive rate (**TPR**) = true positives/(true positives + false negatives), and

false positive rate (**FPR**) = false positives/(true positives + false positives).

## Authors' contributions

GB conceived the study. YQS designed, developed and implemented the methods. GB participated in the design and supervised the process. YQS drafted the manuscript. Both authors approved the final manuscript.

## Supplementary Material

Additional file 2 This file contains scripts for the online server YimLOC. Please note that there scripts only codes for the ready-to-use STACK-mem-DT described in the main text. The scripts do not provide the training process. Click here for file

Additional file 1 This file contains figures and tables depicting the performance of different integration methods on *Arabidopsis *data, human data, a non-overlapping subset of yeast data, and three more stringent datasets. The results were obtained in the same way as for the yeast data. This file also contains a table showing the range of numerical predictions from individual LOC-tools. **Figure S1 – Prediction performance of individual and integrated tools on *Arabidopsis *mitochondrial proteins**. **Filled symbols**: individual LOC-tools; **Dots**: voting groups (tools integrated by majority-win voting); **Open symbols**: decision trees. The desired results are located in the top left of the plot area, representing high true positive rate and low false positive rate. **Figure S2 – Prediction performance of individual and integrated tools on human mitochondrial proteins**. **Filled symbols**: individual LOC-tools; **Dots**: voting groups (tools integrated by majority-win voting); **Open symbols**: decision trees. The desired results are located in the top left of the plot area, representing high true positive rate and low false positive rate. **Figure S3 – Prediction performance of individual and integrated tools on yeast data which does not overlap with the training data of any individual LOC-tool**. **Filled symbols**: individual LOC-tools; **Dots**: voting groups (tools integrated by majority-win voting); **Open symbols**: decision trees. The desired results are located in the top left of the plot area, representing high true positive rate and low false positive rate. Click here for file
