# Supplementary material for: 'Unite and conquer': enhanced prediction of protein subcellular localization by integrating multiple specialized tools
Source: BMC Bioinformatics. 2007 Oct 29;8:420. doi: 10.1186/1471-2105-8-420 (PMC2176073; doi:10.1186/1471-2105-8-420)
Supplement: Additional file 2 — This file contains scripts for the online server YimLOC. Please note that there scripts only codes for the ready-to-use STACK-mem-DT described in the main text. The scripts do not provide the training process. [file 1471-2105-8-420-S2.pdf]

Script for YimLOC-yeast

```
# This script is the implementation of STACK-mem-DT trained by yeast data. Please note that it is only for the exemplification
# purpose and does not provide the training process described in the text.

# This script reads an input file (supplied with -i) with comma-delimited predictions from
# individual LOC-tools, and outputs the prediction by STACK-mem-DT

#!/usr/bin/perl -w

# Usage: program -i inputfile
# This program reads a input file (supplied with -i) with comma-delimited

# Sample input
# T,T,T,T,T,T,T,T,T,0,0,1,0                    # no ID
# P53665,T,F,T,F,F,T,F,F,T,0,0,0,0        # with ID


use strict;

# Get command-line options
use Getopt::Std;
use vars qw( $opt_i );
getopts "i:";
die "Usage: $0 -i inputfile\n"
    unless defined $opt_i;

# Open input and output files
open (R,"<$opt_i") || die "Cannot read from inputfile '$opt_i': $!\n";

# Read the input file, line by line
while (my $line = <R>)
{
```

```
        $line =~ s/\s+//g; # no space needed
        next if $line eq ""; # skip blank lines

        # Verification of format
        die "Incorrect line in file, please verify input format. Faulty line:\n$line\n"
           if $line !~ m/,/ || $line =~ m/([^a-zA-Z0-9,_\-\.\?])+/;

        # Split components;
        my @comps =split(/,/,$line);

        if ( $comps[0] eq "T" || $comps[0] eq "F" )
           {
                   unshift(@comps, "(ID not provided)");
        }

        # Give a name to each component
        my ($id,
           #   1         2          3        4          5          6         7          8           9
           $targetP, $subloc, $Ptarget, $sherloc, $predotar, $mitoprot, $cello, $pprowler, $PASUB, # T or F
           #    10        11
           $Phobius, $tmhmm                                                            # numbers

          ) = @comps;


# Decision tree

           my $mtp=""; # result from MTP-DT

           if ($cello eq "T")
           {
                   if ($PASUB eq "T" || $PASUB eq "N" )
                   {
                           print "$id is a mitochondrial protein.\n";
```

```
			next;
		}
		elsif ($PASUB eq "F")
		{
			if ($Ptarget eq "T")
			{
				print "$id is a mitochondrial protein.\n";
				next;
			}
			elsif ($Ptarget eq "F")
			{
				print "$id is not a mitochondrial protein.\n";
				next;
		}
			else
			{
				print "$id: No result. pTARGET prediction is required.\n";
				next;
			}
		}
		else
		{
			print "$id: No result. PASUB prediction is required.\n";
			next;
		}

	}
	elsif ($cello eq "F")
	{
		if ($predotar eq "T")
		{
			$mtp="T";
		}
		elsif ($predotar eq "F")
```

```
{
	if ($mitoprot eq "T")
	{
		if ($pprowler eq "T")
		{
			$mtp="T";
		}
		elsif ($pprowler eq "F")
		{
			$mtp="F";
		}
		else
		{
			print "$id: No result. PProwler prediction is required.\n";
			next;
		}
	}
	elsif ($mitoprot eq "F")
	{
		$mtp="F";
}
	else
	{
		print "$id: No result. MitoProt prediction is required.\n";
		next;
	}
}
else
{
	print "$id: No result. Predotar prediction is required.\n";
	next;
}
if ($mtp eq "T")
{
```

```
		if ($tmhmm eq "")
		{
			print "$id: No result. TMHMM prediction is required.\n";
			next;
		}
	if ($tmhmm <=5)
		{
			print "$id is a mitochondrial protein.\n";
			next;
	}
		elsif ($tmhmm> 5)
		{
			if ($Phobius eq "")
			{
				print "$id: No result. Phobius prediction is required.\n";
				next;
			}
			if ($Phobius<=8)
			{
				print "$id is a mitochondrial protein.\n";
				next;
			}
			elsif ($Phobius>8)
			{
				print "$id is not a mitochondrial protein.\n";
				next;
			}
		}
	}
	elsif ($mtp eq "F")
	{
		if ($sherloc eq "T")
		{
			if ($Ptarget eq "T")
```

```
		{
			print "$id is a mitochondrial protein.\n";
			next;
		}
		elsif ($Ptarget eq "F")
		{
			print "$id is not a mitochondrial protein.\n";
			next;
		}
		else
		{
			print "$id: No result. pTARGET prediction is required.\n";
			next;
		}
	}
	elsif ($sherloc eq "F")
	{
		if ($Ptarget eq "T")
		{
			if ($PASUB eq "T")
			{
				print "$id is a mitochondrial protein.\n";
				next;
			}
			elsif ($PASUB eq "F" || $PASUB eq "N")
			{
				print "$id is not a mitochondrial protein.\n";
				next;
			}
			else
			{
				print "$id: No result. PASUB prediction is required.\n";
				next;
			}
```

```
}
elsif ($Ptarget eq "F")
{
	if ($PASUB eq "T")
	{
		if ($Phobius eq "")
		{
			print "$id: No result. Phobius prediction is required.\n";
			next;
		}
		if ($Phobius <=5)
		{
			if ($Phobius <=0)
			{
				print "$id is not a mitochondrial protein.\n";
				next;
			}
			elsif ($Phobius >0)
			{
				print "$id is a mitochondrial protein.\n";
				next;
			}
		}
		elsif ($Phobius >5)
		{
			print "$id is not a mitochondrial protein.\n";
			next;
		}
	}
	elsif ($PASUB eq "F" || $PASUB eq "N")
	{
		print "$id is not a mitochondrial protein.\n";
		next;
	}
```

```
                    else
                    {
                        print "$id: No result. PASUB prediction is required.\n";
                        next;
                    }
                }
                else
                {
                    print "$id: No result. pTARGET prediction is required.\n";
                    next;
                }
            }
            else
            {
                print "$id: No result. SherLoc prediction is required.\n";
                next;
            }
        } # end of (elsif MTP eq F); no final else.
    }
    else
    {# CELLO is neither T or F
        print "$id: ", "No result. CELLO prediction is required.\n";
        next;
    }
}  # For each line in input

# Finish everything
close (R);
```

Script for YimLOC-Arabidopsis

This script is the implementation of STACK-mem-DT trained by *Arabidopsis* data. Please note that it is only for the exemplification

```
# purpose and does not provide the training process described in the text.

# This script reads an input file (supplied with -i) with comma-delimited predictions from
# individual LOC-tools, and outputs the prediction by STACK-mem-DT


#!/usr/bin/perl -w

# Usage: program -i inputfile
# This program reads a input file (supplied with -i) with comma-delimited

# Sample input
# T,T,T,T,T,T,T,T,T,0,0,1,0                    # no ID
# P53665,T,F,T,F,F,T,F,F,T,0,0,0,0        # with ID


use strict;

# Get command-line options
use Getopt::Std;
use vars qw( $opt_i );
getopts "i:";
die "Usage: $0 -i inputfile\n"
    unless defined $opt_i;

# Open input and output files
open (R,"<$opt_i") || die "Cannot read from inputfile '$opt_i': $!\n";

# Read the input file, line by line
while (my $line = <R>)
{

        $line =~ s/\s+//g; # no space needed
        next if $line eq ""; # skip blank lines
```

```
# Verification of format
die "Incorrect line in file, please verify input format. Faulty line:\n$line\n"
   if $line !~ m/,/ || $line =~ m/([^a-zA-Z0-9,_\-\.\?])+/;

# Split components;
my @comps =split(/,/,$line);

if ( $comps[0] eq "T" || $comps[0] eq "F" )
{
   #unshift(@comps, ""); # reinsert the missing ID as "ID not provided"
   unshift(@comps, "(ID not provided)");
}

# Give a name to each component
my ($id,
   #   1          2          3        4          5          6           7       8          9
   $targetP, $subloc, $Ptarget, $sherloc, $predotar, $mitoprot, $cello, $pprowler, $PASUB, # T or F
   #    10          11          12          13
   $Phobius, $tmhmm,   $HMMTOP,   $SOSUI                                             # numbers

  ) = @comps;


   # Decision tree
   my $mtp="";
   if ($cello eq "T")
   {print $id, " is a mitochondrial protein.\n"; next;}
   elsif ($cello eq "F")
   {
         if ($predotar eq "T")
         {
               if ($pprowler eq "T")
               {$mtp="T";}
```

```
		elsif ($pprowler eq "F")
		{
			if ($targetP eq "T")
			{$mtp="T";}
			elsif ($targetP eq "F")
			{$mtp="F";}
			else
			{print $id, ": ", "No result. TargetP prediction is required.\n";next;}
		}
		else
		{print $id, ": ", "No result. PProwler prediction is required.\n";next;}
	}
	elsif ($predotar eq "F")
	{$mtp="F";}
	else
	{print $id, ": ", "No result. Predotar prediction is required.\n";next;}
	if ($mtp eq "T")
	{
		if ($SOSUI eq "")
		{print $id, ": ", "No result. SOSUI prediction is required.\n";next;}
		if ($SOSUI<=0)
		{print $id, " is a mitochondrial protein.\n"; next;}
		elsif ($SOSUI>0)
		{print $id, " is not a mitochondrial protein.\n"; next;}

	}
	elsif ($mtp eq "F")
	{
		if ($PASUB eq "T")
		{print $id, " is a mitochondrial protein.\n"; next;}
		elsif ($PASUB eq "F" || $PASUB eq "N" )
		{print $id, " is not a mitochondrial protein.\n"; next;}
		else
		{print $id, ": ", "No result. PASUB prediction is required.\n";next;}
```

```
                }
        }
        else
        {print $id, ": ", "No result. CELLO prediction is required.\n";next;}

}# For each line in input

# Finish everything
close (R);
close (A);
```

Script for YimLOC-human

```
# This script is the implementation of STACK-mem-DT trained by human data. Please note that it is only for the exemplification
# purpose and does not provide the training process described in the text.

# This script reads an input file (supplied with -i) with comma-delimited predictions from
# individual LOC-tools, and outputs the prediction by STACK-mem-DT



# Usage: program -i inputfile
# This program reads a input file (supplied with -i) with comma-delimited

# Sample input
# T,T,T,T,T,T,T,T,T,0,0,1,0                 # no ID
# P53665,T,F,T,F,F,T,F,F,T,0,0,0,0          # with ID

#!/usr/bin/perl -w


use strict;
```

```
# Get command-line options
use Getopt::Std;
use vars qw( $opt_i );
getopts "i:";
die "Usage: $0 -i inputfile\n"
    unless defined $opt_i;

# Open input and output files
open (R,"<$opt_i") || die "Cannot read from inputfile '$opt_i': $!\n";

# Read the input file, line by line
while (my $line = <R>)
{
        $line =~ s/\s+//g; # no space needed
        next if $line eq ""; # skip blank lines

        # Verification of format
        die "Incorrect line in file, please verify input format. Faulty line:\n$line\n"
            if $line !~ m/,/ || $line =~ m/([^a-zA-Z0-9,_\-\.\?])+/;

        # Split components;
        my @comps =split(/,/,$line);

        if ( $comps[0] eq "T" || $comps[0] eq "F" ) {
            #unshift(@comps, ""); # reinsert the missing ID as "ID not provided"
            unshift(@comps, "(ID not provided)");
        }

        # Give a name to each component
        my ($id,
            #   1          2          3         4          5           6          7        8          9
            $targetP, $subloc, $Ptarget, $sherloc, $predotar, $mitoprot, $cello, $pprowler, $PASUB  # T or F
           ) = @comps;
```

```
# Decision tree
my $mtp=""; # result from MTP-DT

$mtp="";
if ($cello eq "T")
{
	if ($sherloc eq "T")
	{print $id, " is a mitochondrial protein.\n"; next;}
	elsif ($sherloc eq "F")
	{
		if ($PASUB eq "T")
		{print $id, " is a mitochondrial protein.\n"; next;}
		elsif ($PASUB eq "F" || $PASUB eq "N" )
		{print $id, " is not a mitochondrial protein.\n"; next;}
		else
		{print $id, ": ", "No result. PASUB prediction is required.\n";next;}
	}
	else
	{print $id, ": ", "SherLoc prediction result is required.\n";next;}
}
elsif ($cello eq "F")
{
	if ($Ptarget eq "T")
	{
		if ($PASUB eq "T")
		{print $id, " is a mitochondrial protein.\n"; next;}
		elsif ($PASUB eq "F")
		{print $id, " is not a mitochondrial protein.\n"; next;}
		elsif ($PASUB eq "N")
		{
			if ($sherloc eq "T")
			{print $id, " is a mitochondrial protein.\n"; next;}
```

```
				elsif ($sherloc eq "F")
				{print $id, " is not a mitochondrial protein.\n"; next;}
				else
				{print $id, ": ", "No result. SherLoc prediction is required.\n";next;}
			}
			else
			{print $id, ": ", "No result. PASUB prediction is required.\n";next;}
		}
		elsif ($Ptarget eq "F")
		{
			if ($PASUB eq "T")
			{
				if ($sherloc eq "T")
				{print $id, " is a mitochondrial protein.\n"; next;}
				elsif ($sherloc eq "F")
				{
					if ($subloc eq "T")
					{print $id, " is a mitochondrial protein.\n"; next;}
					elsif ($subloc eq "F")
					{print $id, " is not a mitochondrial protein.\n"; next;}
					else
					{print $id, ": ", "No result. Subloc prediction is required.\n";next;}
				}
				else
				{print $id, ": ", "No result. SherLoc prediction is required.\n";next;}
			}
			elsif ($PASUB eq "F" || $PASUB eq "N")
			{print $id, " is not a mitochondrial protein.\n"; next;}
			else
			{print $id, ": ","No result. PASUB prediction is required.\n";next;}
		}
		else
		{print $id, ": ", "No result. pTARGET prediction is required.\n";next;}
	}
```

```
        else
        {print $id, ": ", "No result. CELLO prediction is required.\n";next;}

}# For each line in input

# Finish everything
close (R);
close (A);
```
