# Supplementary material for: 'Unite and conquer': enhanced prediction of protein subcellular localization by integrating multiple specialized tools
Source: BMC Bioinformatics. 2007 Oct 29;8:420. doi: 10.1186/1471-2105-8-420 (PMC2176073; doi:10.1186/1471-2105-8-420)
Supplement: Additional file 1 — This file contains figures and tables depicting the performance of different integration methods on Arabidopsis data, human data, a non-overlapping subset of yeast data, and three more stringent datasets. The results were obtained in the same way as for the yeast data. This file also contains a table showing the range of numerical predictions from individual LOC-tools. Figure S1 – Prediction performance of individual and integrated tools on Arabidopsis mitochondrial proteins. Filled symbols: individual LOC-tools; Dots: voting groups (tools integrated by majority-win voting); Open symbols: decision trees. The desired results are located in the top left of the plot area, representing high true positive rate and low false positive rate. Figure S2 – Prediction performance of individual and integrated tools on human mitochondrial proteins. Filled symbols: individual LOC-tools; Dots: voting groups (tools integrated by majority-win voting); Open symbols: decision trees. The desired results are located in the top left of the plot area, representing high true positive rate and low false positive rate. Figure S3 – Prediction performance of individual and integrated tools on yeast data which does not overlap with the training data of any individual LOC-tool. Filled symbols: individual LOC-tools; Dots: voting groups (tools integrated by majority-win voting); Open symbols: decision trees. The desired results are located in the top left of the plot area, representing high true positive rate and low false positive rate. [file 1471-2105-8-420-S1.doc]

**Figure S1 - Prediction performance of individual and integrated tools on *Arabidopsis* mitochondrial proteins.**

**Filled symbols**: individual LOC-tools; **Dots**: voting groups (tools integrated by majority-win voting); **Open symbols**: decision trees. The desired results are located in the top left of the plot area, representing high true positive rate and low false positive rate.

**Figure S2 - Prediction performance of individual and integrated tools on human mitochondrial proteins.**

**Filled symbols**: individual LOC-tools; **Dots**: voting groups (tools integrated by majority-win voting); **Open symbols**: decision trees. The desired results are located in the top left of the plot area, representing high true positive rate and low false positive rate.

**Figure S3 - Prediction performance of individual and integrated tools on yeast data without overlap with the training data of any individual LOC-tool.**

**Filled symbols**: individual LOC-tools; **Dots**: voting groups (tools integrated by majority-win voting); **Open symbols**: decision trees. The desired results are located in the top left of the plot area, representing high true positive rate and low false positive rate.

Table S1 The number of predicted classes and the range of numerical prediction values from individual LOC-tools

|  | TargetP | Subloc | pTARGET | SherLoc | Predotar | MitoProt | CELLO | PProwler | PASUB |
| --- | --- | --- | --- | --- | --- | --- | --- | --- | --- |
| **Number of classes** | 4 | 10 | 9 | 9 | 4 | 2 | 12 | 4 | 10 |
| **Value range** | 1-5 | 1-10 | 1%-100% | 0-1 | 0-1 | 0-1 | >0 | 0-1 | 0-1 |
| **Value of most reliable prediction** | 1 | 10 | 100% | 1 | 1 | 1 | The higher the better | 1 | 1 |

Table S2 Number of instances in each dataset, after being clustered at threshold of 80% sequence identity and 25% sequence identity

|  | Yeast | | *Arabidopsis* | | Human | |
| --- | --- | --- | --- | --- | --- | --- |
| Threshold at clustering | 80% | 25% | 80% | 25% | 80% | 25% |
| Mitochondrial proteins | 503 | 446 | 193 | 158 | 353 | 290 |
| Non-mitochondrial proteins | 872 | 781 | 608 | 383 | 2679 | 1505 |
| Total | 1375 | 1227 | 802 | 541 | 3032 | 1795 |

Table S3 Performance^1^ of the best predictors for the three different prediction schemes (for dataset clustered at threshold of 25% identity)

| **Classes^2^** | | **Individual tool (PASUB)** | | | **Combination of tools by voting^3^** | | | **Decision tree classifier**  **(STACK-mem-DT)** | | |
| --- | --- | --- | --- | --- | --- | --- | --- | --- | --- | --- |
|  |  | TPR | FPR | ACC | TPR | FPR | ACC | TPR | FPR | ACC |
| Yeast | Mit | 0.72 | 0.05 | 0.69 | 0.73 | 0.04 | 0.89 | 0.85 | 0.06 | 0.93 |
|  | Non | 0.67 | 0.06 |  | 0.98 | 0.13 |  | 0.97 | 0.08 |  |
| *Arabidopsis* | Mit | 0.73 | 0.10 | 0.82 | 0.66 | 0.04 | 0.89 | 0.84 | 0.11 | 0.92 |
|  | Non | 0.85 | 0.06 |  | 0.98 | 0.12 |  | 0.96 | 0.07 |  |
| Human | Mit | 0.86 | 0.08 | 0.74 | 0.86 | 0.03 | 0.97 | 0.91 | 0.03 | 0.98 |
|  | Non | 0.72 | 0.02 |  | 0.99 | 0.03 |  | 0.99 | 0.02 |  |

^1^ TPR: true positive rate; FPR: false positive rate; ACC: accuracy (all correctly predicted instances / all instances)

^2^ Mit: mitochondrial proteins; Non: proteins of other subcellular locations

^3^ The best combination of tools is pTARGET+Mitoprot+CELLO for yeast data, PASUB+Sherloc+CELLO for *Arabidopsis* data, and pTARGET +SherLoc+ PASUB+subloc+Mitoprot for human data
